# A Case of 14-Year-Old Male with Fibroma of Tendon Sheath of the Hand with Novel Chromosomal Translocation 4;10

**DOI:** 10.1155/2019/3514013

**Published:** 2019-09-17

**Authors:** Aaron Rubinstein, Valerie Fitzhugh, Irfan Ahmed, Michael Vosbikian

**Affiliations:** ^1^Department of Orthopaedic Surgery, Rutgers New Jersey Medical School, New Jersey, USA; ^2^Department of Pathology, Rutgers New Jersey Medical School, New Jersey, USA

## Abstract

Fibroma of tendon sheath (FTS) is an uncommonly encountered soft tissue mass, which is morphologically distinct from the more commonly seen giant cell tumor of tendon sheath (GCTTS). Initially described in 1936, FTS is typically a slow growing, painless, firm mass with a predilection for the upper extremity, frequently involving the hand. Cases of associated triggering or compression neuropathies have been described when underlying tendons or nerves are affected. Currently, the literature on FTS is sparse and largely limited to case reports. More recently, few reports of cytogenetic analysis on FTS have been reported in the literature. Cellular and chromosomal analysis of FTS tissue revealed chromosomal translocations with yet unknown clinical significance. Here, we present a case report of FTS in a 14-year-old male with a painless enlarging mass on the palmar side of the left hand treated by excision. Subsequent karyotypic analysis revealed a chromosomal translocation t(4;10) (p16;q24), add (10)(q22)[24]. To our knowledge, this is the first description of this chromosomal aberration in the literature.

## 1. Introduction

Fibroma of tendon sheath (FTS) is an uncommonly encountered soft tissue mass, which is morphologically distinct from the more commonly seen giant cell tumor of tendon sheath (GCTTS). Initially described by Geschickter and Copeland in 1936, FTS is typically a slow growing, painless, firm mass with a predilection for the upper extremity [1]. Chung and Enzinger subsequently reviewed 138 cases of such tumors and noted a male to female predilection with a median age of 31 years. The hand and fingers were involved in 81% of the cases with the thumb most commonly affected [2].

The literature on FTS is sparse and largely limited to case reports or small series [3–7]. Commonly, patients present with a painless, minimally tender solitary mass. However, cases of associated triggering or compression neuropathies have been described when underlying tendons or nerves are affected [2–7]. Recurrence rates vary and, although rare in many case reports, have been recorded up to 24% in larger series [2–6].

More recently, few reports of cytogenetic analysis on FTS have been reported in the literature. One case of FTS identified a clonal chromosomal abnormality t(9;11) (p24;q13-14), while another identified a cellular abnormality t(2;11)(q31-32;q12) in half the cells sampled [8, 9]. The clinical significance of these translocations has yet to be fully elucidated.

Here, we present a case report of FTS in a 14-year-old male with a painless enlarging mass of the palmar side of the left hand treated by excision. Subsequent karyotypic analysis revealed a novel chromosomal translocation t(4;10) (p16;q24), add (10)(q22)[24].

## 2. Case Report

**Figure 1:**
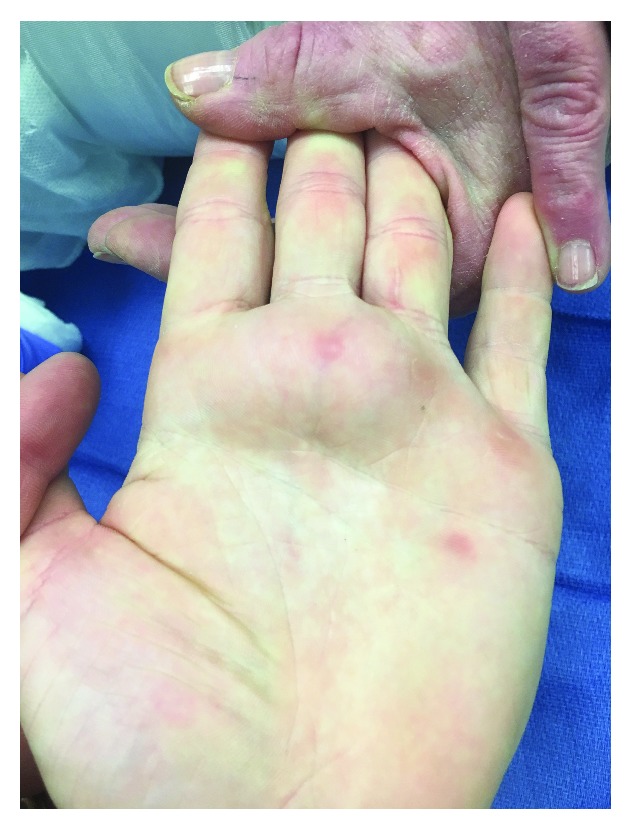
Clinical preoperative images of the patient's hand with volar-based mass spanning the second to fourth metacarpal heads.

A 14-year-old right-hand-dominant Caucasian male presented with a history for several years of a slowly enlarging mass of the left palm centered about the third metacarpal head (Figure 1). The mass was not associated with pain except when batting during baseball. He denied any sensory deficits or motor weakness. Physical examination demonstrated a soft, fixed mass about the palmar aspect of the left hand extending from the region of the second to the fourth metacarpal heads. His neurologic assessment was unremarkable.

**Figure 2:**
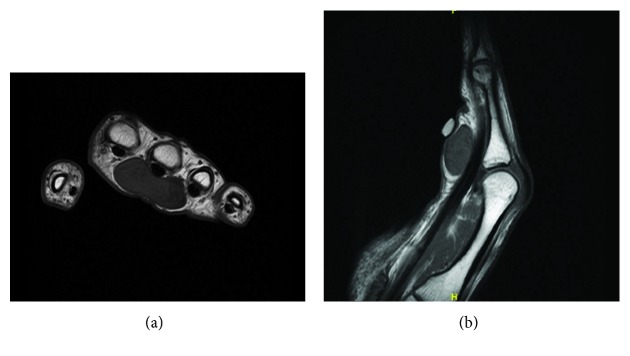
(a, b) Select preoperative T1 axial and T1 sagittal magnetic resonance images of FTS demonstrating relationship to flexor tendon and neurovascular bundle.

Plain radiographs did not show any abnormalities. Magnetic resonance imaging (MRI) with intravenous gadolinium demonstrated a 3.6 × 1.6 × 2.4 cm well-defined soft tissue mass centered about the third metacarpophalangeal joint abutting the flexor tendon sheath of the third digit (Figure 2). The mass demonstrated mild enhancement with contrast. There was no osseous erosion or joint effusion noted. Based on this, the differential diagnosis included infectious etiology versus soft tissue mass including giant cell tumor of tendon sheath, lipoma, leiomyoma, and neuroma.

**Figure 3:**
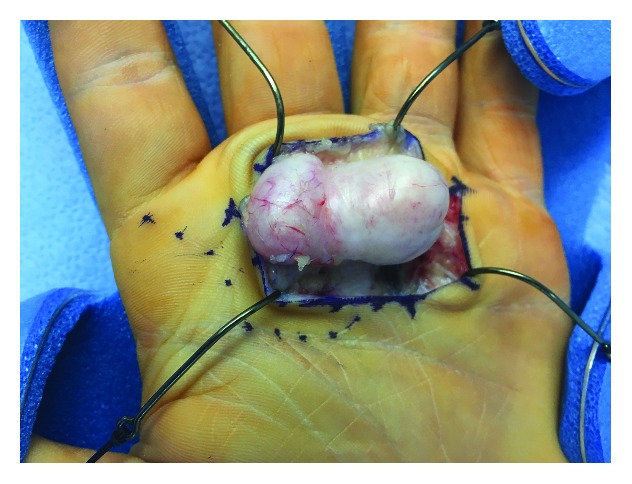
Intraoperative image of well-encapsulated, solitary, solid tan-colored mass shown to be FTS.

**Figure 4:**
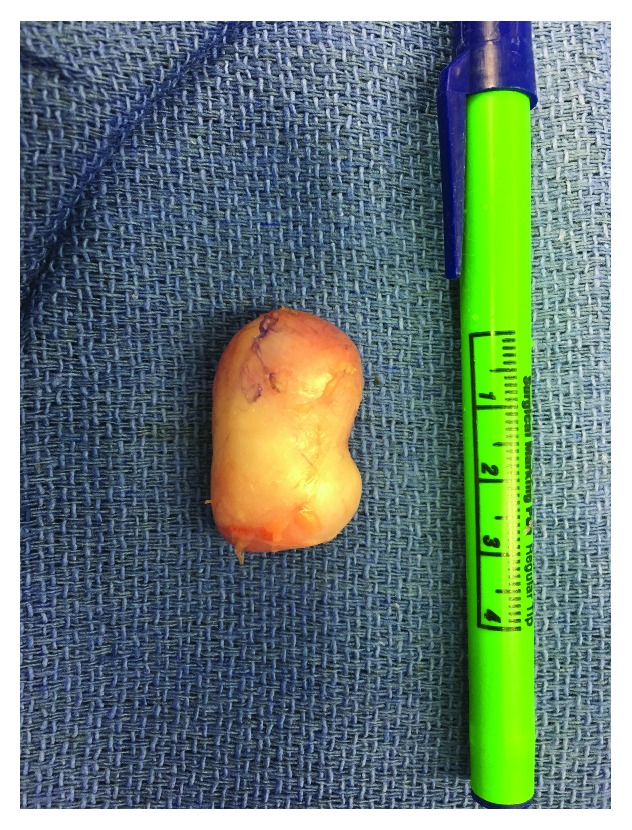
Gross image of excised mass.

The patient underwent excisional biopsy under general anesthesia with tourniquet control after gravity exsanguination. An incision was made in the distal palmar crease overlying the mass, and soft tissue dissection was carried down through the level of the palmar fascia. Intraoperatively, a solitary, solid tan-colored mass was encountered. It was well-encapsulated without communication to any neurovascular or tendinous structure aside from the A1 pulley of the third flexor sheath (Figures 3 and 4). The lesion was removed en bloc. An intraoperative frozen section was performed. The tissue was considered lesional and was deferred for examination by an orthopaedic pathologist.

**Figure 5:**
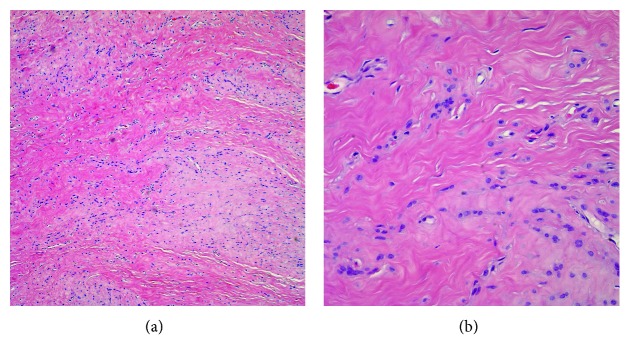
(a) Low-power examination demonstrates a mildly lobulated lesion with tumor cells set in a collagenous matrix. The lesion shows an admixture of hypocellular and hypercellular areas (hematoxylin and eosin stain, magnification 10x). (b) High-power examination demonstrates fibroblasts with basophilic nuclei in a focally hypercellular area. No mitotic figures are identified (hematoxylin and eosin stain, magnification 40x).

Final pathologic examination revealed a 3.8 × 2.5 × 1.3 cm spindle-cell lesion with mild lobulation. The spindle cells resembled fibroblasts, and no nuclear atypia was seen. The lesion was relatively hypocellular without necrosis or hemorrhage. Multinucleate giant cells were not seen (Figure 5). The tumor cells demonstrated focal reactivity to CD34 and smooth muscle actin. Rare cells demonstrated immunoreactivity to calponin. The tumor cells were negative for CD31, Fli-1, CD68, muscle-specific actin (HHF-35), and desmin. Less than 1% of the cells demonstrated proliferation via Ki-67, consistent with a benign process. The histologic and immunohistochemical findings were mostly in keeping with fibroma of tendon sheath. In addition, a portion of the fresh specimen was submitted for karyotype analysis. The results revealed a 46,XY,t(4;10)(p16;q24), add (10)(q22) in twenty-four analyzed cells. All cells analyzed showed a translocation involving chromosomes 4 and 10 and a complex structural rearrangement of the long arm of the other chromosome 10 at band q22.

**Figure 6:**
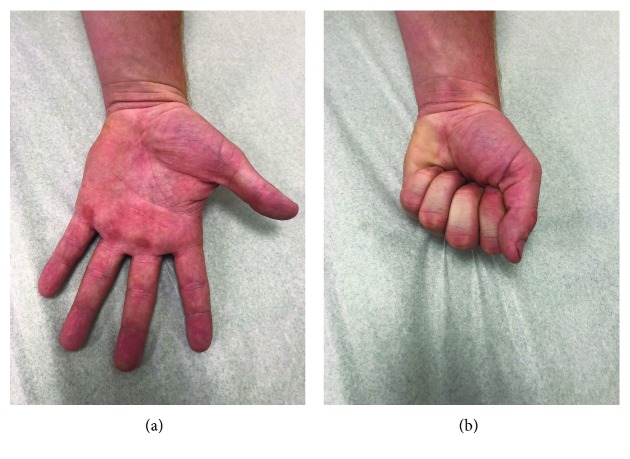
(a, b) Clinical postoperative images of patient 21 months after surgery.

The patient's postoperative course was uneventful, and he returned to baseball activities 6 weeks postsurgery. He is currently at 21-month status postsurgical excision, reports pain-free activity, and exhibits no evidence of local recurrence (Figure 6).

## 3. Discussion

FTS is a relatively uncommon tumor that typically presents as a painless, slow growing, firm nodule in the palm or digits. Typically, FTS is seen in the young to middle-aged population, with some series reporting an up to 3 : 1 male to female ratio [10]. Uncommonly, antecedent trauma is reported [10]. The differential diagnosis should include giant cell tumor, mucinous cyst, lipoma, synovial sarcoma, epidermal cyst, and leiomyoma [3]. Clinically, FTS most commonly behave like giant cell tumors of tendon sheath in that they are firm masses with an intimate anatomic relationship to the tendon sheath. They additionally exhibit similar signal patterns on MR imaging with low to intermediate signal intensity on T1-weighted sequences and intermediate to high signal intensity on T2-weighted images [3, 6, 11, 12].

A combination of history, clinical examination, and magnetic resonance imaging may raise suspicion for this tumor; however, FTS is most effectively differentiated from other soft tissue masses by its histopathologic features. FTS is comprised of fibroblast-like spindle cells with elongated, basophilic nuclei located within a dense collagenous matrix. Generally, there is no cytologic atypia or abundance of mitotic figures [13].

The typical clinical course for FTS is usually uneventful and based primarily on location. Palmar or digital FTS may interfere with gripping activities or sports, as seen in our case. Proximity to neurovascular structures may present as compressive neuropathies with impairments in sensation or strength [5, 7, 14]. Occasionally, triggering of the digits can occur due to the mass's close relationship with flexor tendon sheaths [15]. Typically, surgical excision can be performed on an elective basis. Given the presence of nerve or vascular compromise, however, patients should be counseled on the potential for further clinical worsening if the tumor is not removed in a timely fashion.

Less commonly studied with regard to FTS is the underlying cytogenetic profile of the lesion. In their 1999 case report, Dal Cin et al. describe the first cytogenetic evaluation of a FTS in an attempt to investigate a reactive versus neoplastic origin. Their study identified an identical chromosomal translocation, t(2;11)(q31-32;q12), in half of the sampled cells from a left thumb FTS in a 60-year-old female, suggesting a neoplastic etiology [8]. Nishio et al. subsequently performed a cytogenetic analysis of an FTS surgically removed from the 1^st^ web space of a 38-year-old male. A unique t(9;11)(p24;q13-14) was identified [9].

Our case report identifies a novel cytogenetic rearrangement, t(4;10) (p16;q24), add (10)(q22)[24], in a FTS excised from the palm of a 14-year-old male. As of yet, the clinical relevance of this translocation is unknown. This report provides a starting point for further research into the underlying significance of this abnormality. As seen with anomalies in the chromosome 2q and nodular lesions, specific genetic loci may have implications in the development of benign proliferations [8]. Our study, in turn, may function as a foundation for a novel direction in molecular research to elucidate the pathogenesis of this tumor.

## References

[1] Geschickter C. F., Copeland M. M. (1949). *Tumors of Bone*.

[2] Chung E. B., Enzinger F. M. (1979). Fibroma of tendon sheath. *Cancer*.

[3] Maeda K., Setsu N., Kato Y., Kawai A., Kobayashi E. (2017). Fibroma of tendon sheath presenting limited flexion of the fingers. *Case Reports in Orthopedics*.

[4] Greene T. L., Strickland J. W. (1984). Fibroma of tendon sheath. *The Journal of Hand Surgery*.

[5] Wang B., Zhang J., Li G., Zhang Z. (2016). Fibroma of a tendon sheath causing Guyon’s canal syndrome: case report. *Journal of Plastic Surgery and Hand Surgery*.

[6] Al-Qattan M. M. (2014). Fibroma of tendon sheath of the hand: a series of 20 patients with 23 tumours. *The Journal of Hand Surgery (European Volume)*.

[7] Capkin S., Kaleli T. (2019). Superficial radial nerve compression due to fibroma of the brachioradialis tendon sheath: a case report. *Acta Orthopaedica et Traumatologica Turcica*.

[8] Dal Cin P., Sciot R., De Smet L., Van den Berghe H. (1998). Translocation 2;11 in a fibroma of tendon sheath. *Histopathology*.

[9] Nishio J., Iwasaki H., Nagatomo M., Naito M. (2014). Fibroma of tendon sheath with 11q rearrangements. *Anticancer Research*.

[10] Pulitzer D. R., Martin P. C., Reed R. J. (1989). Fibroma of tendon sheath. A clinicopathologic study of 32 cases. *The American Journal of Surgical Pathology*.

[11] Fox M. G., Kransdorf M. J., Bancroft L. W., Peterson J. J., Flemming D. J. (2003). MR imaging of fibroma of the tendon sheath. *American Journal of Roentgenology*.

[12] Sundaram M., McGuire M. H., Schajowicz F. (1987). Soft-tissue masses: histologic basis for decreased signal (short T2) on T2-weighted MR images. *American Journal of Roentgenology*.

[13] Requena L. K., Heinz (2015). Chapter 11. Fibroma of tendon sheath. *Cutaneous Soft Tissue Tumors*.

[14] Evangelisti S., Reale V. F. (1992). Fibroma of tendon sheath as a cause of carpal tunnel syndrome. *The Journal of Hand Surgery*.

[15] Millon S. J., Bush D. C., Garbes A. D. (1994). Fibroma of tendon sheath in the hand. *The Journal of Hand Surgery*.

